# Association of Toxoplasmosis and COVID-19 in a Mexican Population

**DOI:** 10.3390/microorganisms11061441

**Published:** 2023-05-30

**Authors:** María de la Luz Galván-Ramírez, Angel Gustavo Salas-Lais, José Esteban Muñoz-Medina, Larissa Fernandes-Matano, Laura Rocío Rodríguez Pérez, Karen Franco de León

**Affiliations:** 1 Departamento de Microbiología y Patología, Centro Universitario de Ciencias de la Salud, Universidad de Guadalajara, Guadalajara 44340, Mexico; rocio2427@hotmail.com (L.R.R.P.); karen.franco2307@alumnos.udg.mx (K.F.d.L.); 2 Coordinación de Calidad de Insumos y Laboratorios Especializados, Instituto Mexicano del Seguro Social, Ciudad de Mexico 07760, Mexico; salas_lais@yahoo.com.mx (A.G.S.-L.);

**Keywords:** toxoplasmosis, COVID-19, coinfection, prevalence, antibodies

## Abstract

SARS-CoV-2 is the causal agent of COVID-19; the first report of SARS-CoV-2 infection was in December 2019 in Wuhan, China. This virus has since caused the largest pandemic in history, and the number of deaths and infections has been significant. Nevertheless, the development of vaccines has helped to reduce both deaths and infections. Comorbidities such as diabetes, hypertension, heart and lung diseases, and obesity have been identified as additional risk factors for infection and the progression of COVID-19. Additionally, latent toxoplasmosis has been reported to be a risk factor for acquiring COVID-19 in some studies, but other studies have suggested a negative association between these two infections. Furthermore, in patients after vaccination or with COVID-19 and coinfection, an increase in the lethality and mortality of toxoplasmosis has been observed. Therefore, the objective of the current study is to determine the association of toxoplasmosis with COVID-19 in patients diagnosed with COVID-19. Serum samples from 384 patients previously diagnosed with COVID-19 using IgG antibodies against the S1/S2 antigens of SARS-CoV-2 were collected. Subsequently, anti*-Toxoplasma* IgG and IgM antibodies were analyzed with ELISA. Statistical analysis was performed using SPSS Version 20.0 frequencies, percentages, 2 × 2 tables, and the Pearson correlation coefficient. IgG and IgM anti*-Toxoplasma* antibodies were positive in 105/384 (27.34%) and (26/191) 13.6% of patients, respectively. The positivity for both infections was higher in patients aged >40 years old. Subjects who were overweight or obese were mainly positive for both IgG antibodies against S1/S2 SARS-CoV-2 and *Toxoplasma* antibodies. In conclusion, the coinfection rate was 21.7%. The prevalence of S1/S2 SARS-CoV-2 was 308/384 (80.2%), and the percentage of *Toxoplasma* antibodies was 27.34%.

## 1. Introduction

### 1.1. COVID-19

Severe acute respiratory syndrome coronavirus 2 (SARS-CoV-2) is the causal agent of COVID-19 and has caused the largest pandemic in history [1,2]. Prior to the national vaccination campaign against SARS-CoV-2, Mexico reached 33.5% seroprevalence in December 2020 [3]. To date, our country has reported 7,527,885 confirmed cases and 333,449 deaths from COVID-19 [4]. The most frequent symptoms include fever, cough, and fatigue; likewise, the most associated comorbidities are diabetes, hypertension, lung, and cardiovascular diseases [5]. Being overweight, having a high BMI, and obesity have been considered risk factors for COVID-19 and are associated with a poor clinical prognosis. Additionally, evidence suggests that these patients can harbor viruses for a longer time, increasing the contagious period. The viral load in exhalation is higher since the volume of ventilation increases in these patients. In addition, studies have shown that the exhaled viral load of obese and influenza subjects may be higher due to increased ventilation volume and may spread pathogens more easily [6,7].

Obesity carries a greater risk for comorbidities such as hypertension, heart disease, and diabetes, and an association between hypertension and COVID-19 during the pandemic has been reported, with mortality increasing from 7.8 to 33.3% and the risk for the disease being two or three times greater [8,9]. Cardiovascular diseases are closely related to obesity, hypertension, and diabetes [10].

Overweight and obese patients have poor endothelial function, which is worse than that associated with respiratory diseases and can affect patients with COVID-19. Various studies have shown that, compared to healthy people, patients with pre-existing cardiovascular disease are significantly susceptible to contracting SARS-CoV-2 infection. They also present complications during COVID-19 with a high degree of possibility of leading to death [10,11]. In the state of Coahuila in northern Mexico, a retrospective study was conducted on 17,479 patients who presented with COVID-19, of whom 16,385 were survivors (SV), and 1094 did not survive (NS); notably, of the NS group, 10% had diabetes, and 39.9% and 5.4% had arterial hypertension and cardiac disease, respectively [5].

Sociodemographic factors such as age, sex, poverty, hygiene, and ethnic group are all risk factors for acquiring SARS-CoV-2 infection and developing COVID-19 [12]. In Mexico as well as in other developing countries, there exist other aspects to consider alongside COVID-19 evolution; the health conditions of the population before the pandemic, including chronic diseases; the high existence of rural areas and regions of highly vulnerable social groups; and the attention of the health sector at an institutional level before and during the pandemic [12].

The COVID-19 pandemic has presented SARS-CoV-2 variants B.1.1.529 and the Omicron variant that has been highly contagious worldwide, although the severity of the disease has been milder with a less lethal course for patients. On the other hand, more vaccines against these variants have also emerged, which a large number of the world’s population have received at this date, and the outbreaks of COVID-19 have been less deadly worldwide. However, there are few studies on coinfections and their relationship with variants.

Regarding the association of COVID-19 with other pathogens, there are few studies; nevertheless, coinfections with *Aspergillus* spp, *Candida* spp., *Cryptococcus neoformans*, *Legionella pneumophila, Pneumocystis jirovecii* (*carinii*), mucormycosis, HIV, dengue*, Cytomegalovirus* (CMV), Herpes simplex virus (HSV), *Mycobacterium tuberculosis*, *Strongyloides stercoralis, Trypanosoma cruzi*, and malaria have been reported [13,14,15,16,17,18,19].

### 1.2. Toxoplasmosis

*Toxoplasma gondii* (*T. gondii*) is an obligatory intracellular protozoan and the causal agent of toxoplasmosis. *T. gondii* is a successful parasite due to its high capacity to infect humans and animals [20,21]. Regarding transmission, 80% of human infections occur orally through raw or undercooked meat contaminated with cysts or in food irrigated with wastewater contaminated with oocysts [19]. Vertical or congenital toxoplasmosis also occurs through transmission from mother to child. In this particular case, this happens due to the action of the tachyzoite, capable of crossing the placenta, which acts as a biological barrier mechanism, and this parasite is responsible for the destruction of tissues in the brain, eyes, and the placenta itself, decreasing its thickness [22]. Organ transplantation occurs if a donor has a chronic infection and when the recipient, who is under immunosuppressive treatment to avoid rejecting the transplant, receives an infected organ and may suffer a generalized acute illness. Blood transfusion is a transmission risk of *T. gondii* because the cells present in the blood can cause an infection in the host that receives the transfusion. Additionally, direct transmission occurs when working in laboratories with hand injuries or handling contaminated raw meat [19]. Recently, a hypothesis regarding the mechanism of sexual transmission was suggested [23].

### 1.3. Innate Immune Response

The innate immune response is an anatomical barrier, such as the skin, mucosa, and epithelium. On the other hand, a complement cellar contains a series of approximately thirty proteins that perform antigen lysis, and some phagocytic cells, such as macrophages, dendritic cells, and neutrophils, are responsible for phagocytizing and killing the parasite. Natural killer cells recognize and kill infected cells. This innate system relies on pattern recognition receptors (PRRs) that recognize single molecules or structures of the antigen called pathogen-associated molecular patterns (PAMPs). In the PRR group, these are Toll-like receptors (TLRs), which are responsible for detecting PAMPs from extracellular microorganisms and nucleotide-binding oligomerization domain receptors (NLRs), which detect intracellular antigens. TLR receptors are expressed by macrophages and dendritic cells, which fulfill their function as sensor cells and respond directly or produce inflammatory mediators. This is the case with some cytokines such as IL-1, which is related to inflammatory processes, where cytokines such as IL-12 are responsible for the activation of Natural killer (NK) cells and Interferon gamma (IFN-γ) for the activation of macrophages [24]. SARS-CoV-2 and *Toxoplasma* are intracellular pathogens, and in the immune response to both infections, they activate TLRs, such as TLR2, TLR4, and TLR7. This suggests that the elevated production of some stimulated cytokines in toxoplasmosis patients may worsen the severity of coronavirus disease [25].

On the other hand, an adaptive immune response occurs through B and T lymphocytes, which recognize the antigen through specific receptors. This adaptive response can be divided into humoral immunity and cellular immunity. B lymphocytes are involved in humoral immunity, which occurs through antibodies that are generated by contact with the antigen. The isotype of the antibody depends on the presence of some cytokines [24].

T lymphocytes are involved in cellular immunity once they come into contact with an antigen through antigen-presenting cells, such as B lymphocytes, dendritic cells, and macrophages, T lymphocytes CD4^+^ T helper or helper (Th) lymphocytes, and CD8^+^ T cytotoxic (tc) lymphocytes. Helper T lymphocytes can present a type 1 pro-inflammatory profile involving cytokines such as IL-2 and IFN-γ or a type 2 anti-inflammatory profile, such as Interleukins-10,13,and 4 (IL-10, IL-13, and IL-4). Cytotoxic CD8^+^ T lymphocytes destroy cells that are infected by a parasite. Finally, once the B and T lymphocytes are in contact together with the antigen, they can differentiate into memory cells (specific antibodies) [24,25,26].

### 1.4. Epidemiology

Toxoplasmosis is a cosmopolitan disease with a high prevalence, and at least one-third of the global population has come into contact with this parasite. The incidence of infection varies according to climate [27]. The prevalence of toxoplasmosis in different geographical areas is related to latitude and longitude. One study showed that the highest prevalence was in the range of 0–10° (49.4%), followed by ≥50° (26.8%); and for the longitude, the lowest prevalence was in the range of 80–90° (44.2%), followed by 110–120° (7.8%). Regarding climatic parameters, the highest and lowest prevalence rates occurred in regions with mean relative humidity of 80% (46.6%) and <40% (27.0%); an annual precipitation between 1000 and 1500 mm (39.2%) and 250–500 mm (26.8%); and mean annual temperature of 20 × 30 °C (36.5%). Furthermore, undercooked meat is a factor for its higher prevalence in populations with such a diet. A study was conducted in the USA to examine the quantitative microbial hazard from farm to table for domestically produced lamb, with *T. gondii* prevalence in market lambs pooled from 2011. The bradyzoite concentration was determined in cysts with log-linear regression and an exponential dose-response model. The authors found that the average probability of *Toxoplasma* infection per portion of lamb was approximately 6300 new infections per year in the American population [28].

Blood donors also have a higher risk of transmission. In a study on blood donors from Sidi Bel Abbès in western Algeria, the risk prevalence was 47.7% (95% CI: 35.1–54.3). There were significant differences between other African countries in the West, East, and Central Africa, but not in Algerian pregnant women and countries in North Africa [29].

The risk of toxoplasmosis is higher in pregnant women or those who have had an abortion worldwide, with a prevalence of 7% to 51.3%; in women with abnormal pregnancies and who have undergone an abortion, the prevalence rate is 17.5% to 52.3% [27]. In Mexico, the HIV-positive rate is between 18.2% and 44.8% among women who have had an abortion, with a prevalence in high-risk pregnancies of 34.9% and in women who have had a routine abortion of 44.9% [20].

*Toxoplasma* infection in cats depends on the type of meal. Another important factor is whether the cat lives inside or outside the home. A study of *T. gondii* in cats and their owners was carried out in the Bangkok metropolitan area; of the 327 humans included, the prevalence of *Toxoplasma* antibody was low at 6.4% and 7.3% in 315 cats. By contrast, a Mexican study included 59 cat owners. Thirty-eight (64%) of them were positive for IgG anti-*Toxoplasma*. The seropositivity for cats was 70.8% for IgG and 8.3% for IgM [20].

Toxoplasmosis in HIV-positive patients depends on the geographical region. The highest prevalence of *T. gondii* infection in HIV-infected individuals occurs in African countries, and the highest prevalence is in North Sudan, at 75% (62.2–87.8%); in Mexico, it is 48.7% (41.5–55.8%) [20]. The prevalence of *Toxoplasma* infection in patients with schizophrenia varies across the distribution, with the highest prevalence being in Ethiopia at 87.7%; it was also 51.7% in Mexico [20,30].

The coinfection of COVID-19 and toxoplasmosis has been poorly studied with contradictory results [25]. In the Czech Republic, preliminary results found latent toxoplasmosis to be a possible risk factor for COVID-19 [31]. Subsequently, in Iran, in 269 patients with COVID-19, no significant association between chronic toxoplasmosis and COVID-19 was found [32]. Likewise, there are clinical cases of patients with toxoplasmosis after infection or vaccination with COVID-19 who may be at risk of retinochoroiditis [33]. Another study reported an increase in the mortality of patients with toxoplasmosis and schizophrenia [34]. Therefore, the purpose of this study was to determine the association of anti-*Toxoplasma* and IgG antibodies against the S1/S2 SARS-CoV-2 in a Mexican population in the State of Mexico.

## 2. Materials and Methods

### 2.1. Patients

The samples corresponded to people from the State of Mexico (Mexico) who had not received their first dose of the SARS-CoV2 vaccine; samples were collected from 14 May to 27 July 2021 [35]. A total of 384 serum samples from patients who attended Specialized Laboratories, Mexican Social Security Institute, were diagnosed with COVID-19.

$$N=\frac{\;{\left(Z\right)}^{2}\left(p\right)\left(q\right)}{d^{2}}$$

Considering that Z = 1.96 (95% confidence, d = 0.05 (precision), p = 31.2 prevalence of toxoplasmosis referred to the State of Mexico), q = 68.8 (estimated proportion without toxoplasmosis), N = 329.71.

The calculation of the number of samples for COVID-19 was considered with a prevalence of 35.5 according to a previous study, and the number of samples was 351 [3].

A questionnaire was completed by patients to gather information about the following aspects: diabetes, hypertension, autoimmune diseases, respiratory diseases, and cardiac diseases. Sociodemographic and risk factors were as follows: age, sex, and use of alcohol, tobacco, or drugs. These variables were analyzed with positivity for anti-*Toxoplasma* antibodies and COVID-19.

### 2.2. Detection of IgG Antibodies against the S1/S2 Antigens of SARS-CoV-2

IgG antibodies were analyzed using 200 μL of serum and a LIAISON SARS-CoV-2 S1/S2 IgG Kit (Diasorin, Saluggia, Italy; catalog number 3114450) [36]; the tests and Liaison XL equipment were used following the manufacturer’s instructions. Negative and positive controls were included to validate the results. The cut-off values were negative <15 AU/mL and positive ≥15 AU/mL.

### 2.3. Detection of IgG/IgM Antibodies against Toxoplasma gondii

IgG/IgM anti-*T. gondii* (Calbiotech, A Life Science Company, Cajon California, United States, Catalogues No. TX022G and TX024M) titers were determined in all samples by ELISA. These antibodies were used according to the manufacturer’s instructions at room temperature. Briefly, all samples were diluted 1:21. One hundred microliters of the control and calibrator serum and reagent blank were added and incubated for 20 min. Subsequently, the liquid was removed and washed 3 times with a 1× washing buffer. Next, 100 µL of the conjugated enzyme was added and incubated for 20 min. The enzyme conjugate was removed and rewashed. The plate was placed on absorbent paper, and 100 µL of the TMB substrate was added and incubated for 10 min. Then, 100 µL of the stop solution was added, and the plate was read at an absorbance of 450/620 nm. The cut-off value was calculated as follows: calibrator OD x calibrator factor (CF). The Ab (antibody) index of each determination was calculated by dividing the mean values of each sample by the cut-off value.

### 2.4. Statistical Analysis

SPSS version 20.0 and SPSS v. 18 packages from IBM, Los Angeles, were used to perform all statistical analyses. Quantitative variables are included in the ELISA test results. The statistical significance between the two groups (positive vs. negative to IgG, IgM *Toxoplasma* ELISA) for the differences observed between these variables was obtained with 2 × 2 tables and the Pearson (R) correlation coefficient between the ELISA values and other variables.

## 3. Results

### 3.1. Anti-Toxoplasma Antibodies

Among the 384 patients, the ELISA test results for IgG *Toxoplasma* antibodies were negative in 279 patients and positive in 105 patients, corresponding to a prevalence of 27.34%. For the referent of IgM in 191 samples analyzed, 26/191 (13.6%) were positive. The 26 patients that were IgM-positive had high levels of IgM and IgG anti-*Toxoplasma*. The positivity of IgG e IgM antibodies to *Toxoplasma* and SARS-CoV-2 was analyzed with sociodemographic variables alongside clinical and risk factors. For IgM antibodies, no statistically significant differences were found.

### 3.2. IgG Antibodies against the S1/S2 Antigens of SARS-CoV-2

**Figure 1:**
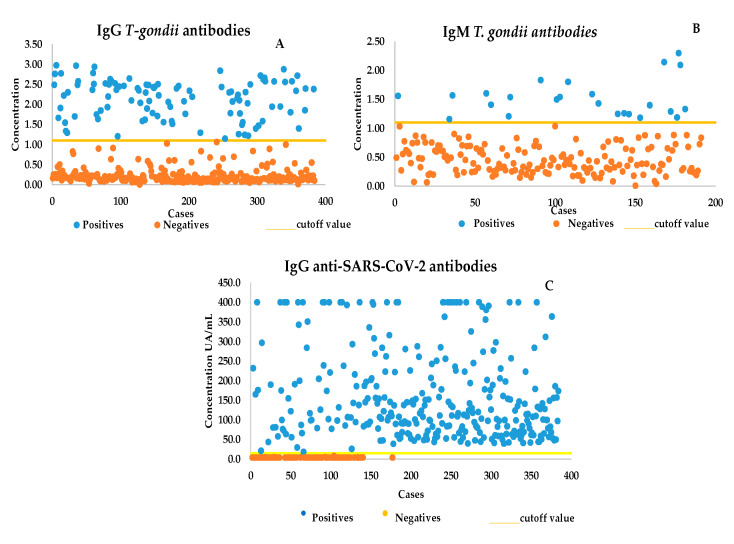
(**A**) The high levels of anti-*Toxoplasma* IgG antibodies. (**B**) Most anti-*Toxoplasma* IgM antibodies were elevated. (**C**) High levels of IgG antibodies against S1/S2 SARS-CoV-2.

IgG anti-SARS-CoV-2 antibodies. IgG-positive 308/384 (80.2%) patients presented elevated levels of anti-SARS-CoV-2 antibodies. A total of 13/26 patients who tested positive for IgM (50%) and 84/384 (10.9%) who were positive for IgG had anti-SARS-CoV-2 antibodies, with a high level of both antibodies (Figure 1).

### 3.3. Sociodemographic Data

The mean age of all patients was 47.31 years with a standard deviation of ±10.22 years; the minimum age was 19 years, and the maximum age was 74. The mean age of the women was 46.64 years with a standard deviation of ±10.53 years, the minimum age was 22 years, and the maximum age was 74 years; in the case of the men, the mean age was 48.20 ± 9.60 years with a minimum age of 19 years and a maximum age of 66 years. See Table 1.

A total of 242 women and 142 men were studied, and IgG antibodies against antigens S1/S2 SARS-CoV-2 positivity was higher in women 196/242 (80.9%) than in men 112/142 (78.87%); however, this difference was not significant. There was also no significant difference in the number of women versus men (*p* = 0.077).

In relation to *Toxoplasma gondii* infection in patients, those most infected were those in the same age group (>40 years) as those infected with COVID-19; when compared with those aged <40 years, a statistically significant difference was found (*p* = 0.001) (Table 2).

Concerning the comorbidities of the patients studied, we analyzed the presence or absence of diabetes, hypertension, respiratory, cardiac, and autoimmune diseases, and the majority of patients who did not have these diseases were highly positive for *Toxoplasma* antibodies. The distribution is shown in (Table 3) as well as SARS-CoV-2 (Table 4).

Positivity for anti-*Toxoplasma* antibodies and IgG antibodies against S1/S2 SARS-CoV-2 and risk factors such as the use of alcohol, tobacco, or drugs and the type of consumption were all compared with the X^2^ test. However, a greater prevalence was found in patients’ of the non-consumer category, though in no case was a statistically significant association found, as shown in Table 5 and Table 6.

The weight of the patients and seropositivity to anti-*Toxoplasma* antibodies were analyzed. A total of 69/384 (18%) patients had a normal weight, 155/384 were overweight, and 160/384 were obese; in total, 15/69 (21.73%) were positive for *Toxoplasma*, and 54/69 (78.2%) were negative. When we compared the normal weight group against the overweight or obese group, we did not find a significant difference (Table 7).

In patients with SARS-CoV-2, 61/69 (88.98%) patients who were not overweight were positive, while 126/155 (81.4%) were in the overweight group. The difference between the normal group and the obesity group was statistically significant (*p* = 0.01; Table 7).

## 4. Discussion

A total of 384 serum samples from patients with IgG antibodies against S1/S2 SARS-CoV-2 were previously analyzed for anti-*T. gondii* antibodies (IgM and IgG ELISA) to determine their coinfection with SARS-CoV-2. This study demonstrated that 105/384 (27.34%) were positive for anti-*T. gondii* IgG. Anti-*T. gondii* IgM was found in 191 patients, and 26/191 (13.6%) were positive.

With respect to coinfection, only 84/384 (21.57%) were positive for the *Toxoplasma* infection and SARS-CoV-2. The prevalence of IgG antibodies was 27.34%, which is similar to other studies in an open population group in Mexico [37]. According to a study in the State of Mexico, the IgG anti-*Toxoplasma* prevalence determined by immunofluorescence ranged from 19.7% to 32.1% [38], and toxoplasmosis in patients with COVID-19 was in the range of that reported for this state. However, in areas with high temperature, humidity, and precipitation, toxoplasmosis prevalence rates were higher; they might also be lower in other regions depending on geographic location, climate, and dietary habits [27,39].

The correlation between *Toxoplasma* and SARS-CoV-2 did not reach statistical significance. However, high levels of IgG antibodies were present in 80% and 100% of patients with IgM positivity for COVID-19. This may be due to acute toxoplasmosis, which was prevalent in patients with COVID-19. Another explanation may be that SARS-CoV-2 infection decreased the number of T cells, NK cells, monocytes, and dendritic cells and lowered the production of IFN-γ, which is essential in the response against *T. gondii*, increasing the risk of acute toxoplasmosis or reactivation. This is consistent with what has been demonstrated in vitro by other authors [2].

Regarding age, the >40-year-old group presented the highest positivity for anti*-Toxoplasma* IgG antibodies (90/271, 33.3%). For IgG anti-SARS-CoV-2 antibodies, 198/271 (73.0%) were positive. This result may be because the prevalence of toxoplasmosis increased with age, and for COVID-19, those over 40 years of age were the most affected; this result was similar to those of other studies [40].

In this study, comorbidities were statistically significant in patients with diabetes and hypertension. However, a higher number of positive patients without hypertension and diabetes with SARS-CoV-2 or latent or acute toxoplasmosis was opposite to that reported at the start of the pandemic. Namely, COVID-19 patients with these comorbidities were initially reported to have a higher risk of fatality [41,42,43]. This discrepancy may be because, to date, more of the population has been infected with SARS-CoV-2, even without these comorbidities.

The number of toxoplasmosis patients without hypertension (35%) who presented anti-*Toxoplasma* IgG antibodies was lower than in patients with this comorbidity (64.8%) or those with heart diseases (99.9%) or diabetes (77.1%). These results are different from a study in Iran where patients with *Toxoplasma* infection were associated with diabetes at 7.5%, hypertension at 12.4%, and heart diseases at 15.5%.

This may be due to the characteristics of the population studied in Mexico, where being overweight and obese are most strongly associated with these comorbidities. On the other hand, another study suggested that chronic toxoplasmosis was a possible risk factor for type 2 DM [44,45].

Being overweight or obese in patients was not significantly associated with *Toxoplasma* seropositivity compared to patients with a normal weight. Patients with SARS-CoV-2 were statistically associated with the obesity group (*p* < 0.01). There could be two explanations for these findings. First, there was a high prevalence of obesity and overweight in the Mexican population and an increased risk in patients with SARS-CoV-2. Other studies have shown a higher prevalence of COVID-19 in those with obesity in the United States (40%) and 6.2% in China [43,44]. In another study, IgG anti-*Toxoplasma* seropositive patients were associated with type 1 and 2 diabetes and obesity [45]. Another possibility is that in patients with obesity, insulin resistance leads to an abnormal T-cell response to pathogens and a deficiency in CD4^+^ T and CD8^+^ T lymphocytes, which is essential for their adaptive immune response against infection [43,44].

## 5. Conclusions

Twenty-two percent of patients had anti-*Toxoplasma* and IgG antibodies against the S1/S2 antigens of SARS-CoV-2, and 50% of patients who were positive for IgM had IgG antibodies against the antigens S1/S2 SARS-CoV-2. Patients aged > 40 years were more likely to have both an S1/S2 SARS-CoV-2 and *Toxoplasma* infection.

Patients who were obese had a 2.4 greater risk of infection with COVID-19.

## Figures and Tables

**Table 1:** Distribution of IgG anti*-Toxoplasma* antibodies by age and sex.

Variable	No.	IgG *T. gondii* +	%	IgG *T. gondii −*	%	X^2^	*p*	OR	95% CI
<40 years old	113	15	14.3	98	85.21	15.5	0.001	0.30	0.16–0.55
>40 years	271	90	33.2	181	66.8				
Total	384	105		279					
Female	242	65	61.9	177	36.6	0.07	0.78	0.93	0.58–1.4
Male	142	40	38.1	102	60.5				

**Table 2:** Distribution IgG antibodies against antigens S1/S2 SARS-CoV-2 by age and sex.

Variable	No.	SARS- CoV-2 +	%	SARS- CoV-2 −	%	X^2^	*p*	OR	95% CI
<40 years old	113	110	97.34	3	2.6	29.6	0.001	13.46	4.6–55.1
>40 years	271	198	73.00	73	26.9				
Total	384	308		73					
Female	242	196	80.9	46	19.1	0.253	0.61	1.143	0.67–1.9
Male	142	112	78.87	30	21.1				

**Table 3:** Distribution of positivity for IgG anti-*Toxoplasma* antibodies and clinical comorbidities.

Variables	Positives		Total	X^2^	*p*	OR	95% CI
	No.	%					
**IgG anti-*Toxoplasma***							
**Diabetes**							
Yes	24	22.9	74	1.19	0.310	1.35	0.74–2.34
No	81	77.1	310				
Total	105		384				
**Hypertension**							
Yes	2	1.9	8	0.022	0.620	0.883	0.175–4.4
No	103	98.1	376				
Total	105		384				
**Respiratory Diseases**							
Yes	2	1.9	110	0.478	0.382	0.583	0.124–2.74
No	103	98.1	373				
Total	105		384				
**Cardiac Diseases**							
Yes	1	1.0	4	0.011	0.91	0.88	0.91–8.60
No	104	99.0	380				
Total	105		384				

**Table 4:** Distribution of IgG antibodies against S1/S2 SARS-CoV-2 positivity and clinical comorbidities.

Variables	Positives						
			Total	X^2^	*p*	OR	95% CI
	No.	%					
**IgG SARS-CoV-2**							
**Diabetes**							
Yes	36	11.7	74	57.1	0.001	0.13	0.075–0.234
No	272	88.3	310				
**Hypertension**							
Yes	57	18.5	109	74.1	0.001	0.105	0.60–0.184
No	251	81.5	275				
Total	308		384				
**Autoimmune Diseases**							
Yes	8	2.6	8				
No	300	97.4	376				
Total	308		384	0.22	0.168	1.25	1.19–1–31
**Respiratory Diseases**							
Yes	11	3.6	11				
No	297	96.4	373				
Total	308		384	2.7	0.08	1.25	1.19–1.32
**Cardiac Diseases**							
Yes * No Total	4 304 308	1.3 98.7	4 308 384	24.5	0.0001	0.17	0.004–0.76

* X^2^ = corrected.

**Table 5:** Seropositivity of IgG anti-*Toxoplasma* and consumption of tobacco, alcohol, and drugs.

Consumption	Number	Positives	%	X^2^	*p*
**Tobacco**					
Mild	46	42	12.6	1.8	0.692
Moderate	16	6	5.7		
Severe	10	4	3.8		
No	312	82	78.1		
**Alcohol**					
Mild	105	29	27.6	0.996	0.89
Moderate	7	1	1.0		
Severe	1	0	0.0		
No	271	75	71.4		
**Drugs**					
No	381	104	99.0	0.55	0.18
Yes	3	1	1.0		

**Table 6:** Seropositivity for IgG antibodies against S1/S2 SARS-CoV-2 and consumption of tobacco, alcohol, and drugs.

Consumption	Number	Positives	%	X^2^	*p*
**Tobacco**					
Mild	46	42	13.6	6.3	0.09
Moderate	16	13	4.2		
Severe	10	6	1.9		
No	312	247	80.2		
**Alcohol**					
Mild	105	81	26.3	1.16	0.70
Moderate	7	6	1.9		
Severe	1	3	0.3		
No	271	220	71.4		
**Drugs**					
No	381	305	99.0	0.74	0.51
Yes	3	3	1.0		

**Table 7:** Seropositivity of anti-*Toxoplasma*, IgG antibodies against S1/S2 SARS-CoV-2 with overweight or obesity.

Variables	Positives			X^2^	*p*	OR	95% CI
	Total	No.	%				
**IgG anti-*Toxoplasma***							
* Normal	69	15	14.3				
Overweight	155	47	44.8	1.7	0.09	0.63	0.32–1.23
Obesity	160	43	41.0	0.67	0.20	0.75	0.3–1.4
Total	384	105					
**IgG antibodies against S1/S2 SARS-CoV-2**							
* Normal	69	61	19.8				
Overweight	155	126	40.9	1.7	0.09	1.7	0.77–4.3
Obesity	160	121	39.3	4.8	0.01	2.4	1.1–5.9
Total	384	308					

* Normal group was compared with the overweight and obese groups.

## Data Availability

Data available upon request from the corresponding author, and public availability is limited due to privacy and ethical constraints.
